# Utilization of Lyotropic Liquid Crystalline Gels for Chronic Wound Management

**DOI:** 10.3390/gels9090738

**Published:** 2023-09-12

**Authors:** Peili Luo, Lei Shu, Zhengwei Huang, Ying Huang, Chuanbin Wu, Xin Pan, Ping Hu

**Affiliations:** 1 College of Pharmacy, Jinan University, Guangzhou 511443, China; stu202235831056lpl@stu2022.jnu.edu.cn (P.L.); shulei@stu2021.jnu.edu.cn (L.S.); chuanbin_wu@126.com (C.W.); inzahu@hotmail.com (P.H.); 2 School of Pharmaceutical Sciences, Sun Yat-Sen University, Guangzhou 510006, China; mercurypan@foxmail.com

**Keywords:** chronic wound, growth factors, healing environment, delivery systems, lyotropic liquid crystalline

## Abstract

Management of chronic wounds is becoming a serious health problem worldwide. To treat chronic wounds, a suitable healing environment and sustained delivery of growth factors must be guaranteed. Different therapies have been applied for the treatment of chronic wounds such as debridement and photodynamic therapy. Among them, growth factors are widely used therapeutic drugs. However, at present, growth factor delivery systems cannot meet the demand of clinical practice; therefore new methods should be developed to meet the emerging need. For this reason, researchers have tried to modify hydrogels through some methods such as chemical synthesis and molecule modifications to enhance their properties. However, there are still a large number of limitations in practical use like byproduct problems, difficulty to industrialize, and instability of growth factor. Moreover, applications of new materials like lyotropic liquid crystalline (LLC) on chronic wounds have emerged as a new trend. The structure of LLC is endowed with many excellent properties including low cost, ordered structure, and excellent loading efficiency. LLC can provide a moist local environment for the wound, and its lattice structure can embed the growth factors in the water channel. Growth factor is released from the high-concentration carrier to the low-concentration release medium, which can be precisely regulated. Therefore, it can provide sustained and stable delivery of growth factors as well as a suitable healing environment for wounds, which is a promising candidate for chronic wound healing and has a broad prospective application. In conclusion, more reliable and applicable drug delivery systems should be designed and tested to improve the therapy and management of chronic wounds.

## 1. Introduction

### 1.1. Chronic Wound

A chronic wound is defined as a wound that cannot be repaired in a normal, timely, and orderly manner to achieve anatomical and functional integrity within 8 weeks [1]. It is generally divided into five common types: pressure ulcer, venous ulcer, arterial ulcer, diabetic ulcer, and traumatic ulcer. Its etiology mainly includes chronic diseases, vascular problems, diabetes, neuropathy, malnutrition, old age, stress, infection, and edema [2]. It has become a major problem in the global medical field due to its high prevalence, long healing cycle, expensive treatment, and serious consequences such as amputation, and even death. In 2017, there were 6.7 million chronic wound patients in the United States, with an annual growth rate of more than 2%. The annual treatment cost is as high as USD 50 billion, and it is expected to increase yearly at a rate of 10% [3].

**Figure 1:**
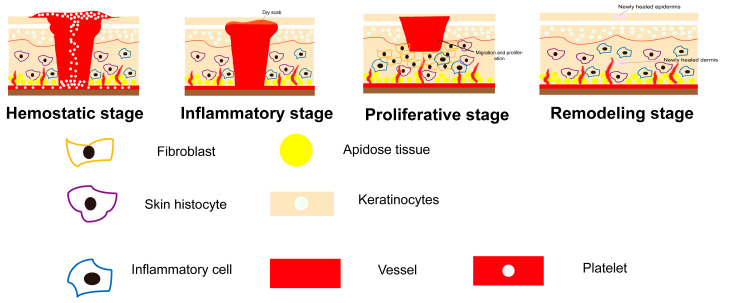
Inflammatory, proliferative, and remodeling stage during wound healing.

Wound healing is an orderly biological process with high complexity and precise regulation. The whole process is divided into four stages (Figure 1): hemostatic stage, inflammatory stage, proliferative stage, and remodeling stage [4,5]. (1) The hemostatic stage begins once blood leaks into the exposed wound and ends within the first minutes to hours of injury. Platelets aggregate and activate subendothelial collagen after the extrinsic clotting cascade is triggered and localized vasoconstriction mediators are released, leading to the formation of a hemostatic plug through the release of cytokines and growth factors. Hemostatic plug can also serve as a provisional matrix for many cell types involved in later stages of wound healing [6]. For non-bleeding chronic wounds, the hemostasis stage is generally omitted, but it is still an inherit stage of healing. (2) In inflammatory stage, platelets aggregate and leukocytes infiltrate into the wound site [7], aiming at building an immune barrier against microorganisms. Neutrophils perform phagocytosis by releasing proteolytic enzymes and oxygen-derived free-radical species to remove bacteria and xenobiotics from wounds [8]. They are removed from the wound before proceeding to the next stage of healing without causing tissue damage or enhancing inflammatory response. Then, macrophages appear in the wound and continue the process of phagocytosis [9]. (3) When it comes to the proliferative phase, fibroblasts play an important role. They proliferate and migrate to the wound, followed by changing into a myofibroblast phenotype and extending themselves to attach to fibronectin and collagen in the extracellular matrix. By synthesizing collagen, they build the integrity and strength of tissues [10]. In addition, endothelial cells and keratinocytes secret growth factors like keratinocyte growth factor 2 (KGF-2) and vascular endothelial growth factor (VEGF), leading to granulation tissue growth, neovascularization, and re-epithelization and promoting wound healing [11]. (4) As the final step, in remodeling stage, synthesis and breakdown of collagen as well as extracellular matrix remodeling take place continuously, which is regulated by inhibitory factors. The process is regulated by a number of factors like platelet-derived growth factor (PDGF), transforming growth factor-β (TGF-β), and fibroblast growth factor (FGF) [12]. Overall, the healing process involves the differentiation, migration, and proliferation of a variety of inflammatory and tissue cells, as well as the interaction between cells and structural proteins, cytokines, and protein kinases [4]. Any stage that hinders wound healing will affect the normal repair of the wound, and eventually form a chronic wound.

### 1.2. Healing Conditions

To treat chronic wounds, besides the treatment of primary diseases, an overall management of the wound is needed, which usually includes drug treatment, routine cleaning, local debridement, and infection control. During the treatment of chronic wounds, the local environment of the wound affects the whole healing process. Sufficient growth factors can promote the formation of granulation tissue and accelerate the re-epithelization process of wound, which is the main factor to prevent chronic wound formation and an important premise to promote wound repair [11]. Therefore, providing a suitable healing environment and stable delivery of growth factors are the keys to treat chronic wounds. The detailed underlying mechanisms are discussed as follows.

#### 1.2.1. Suitable Healing Environment

**Figure 2:**
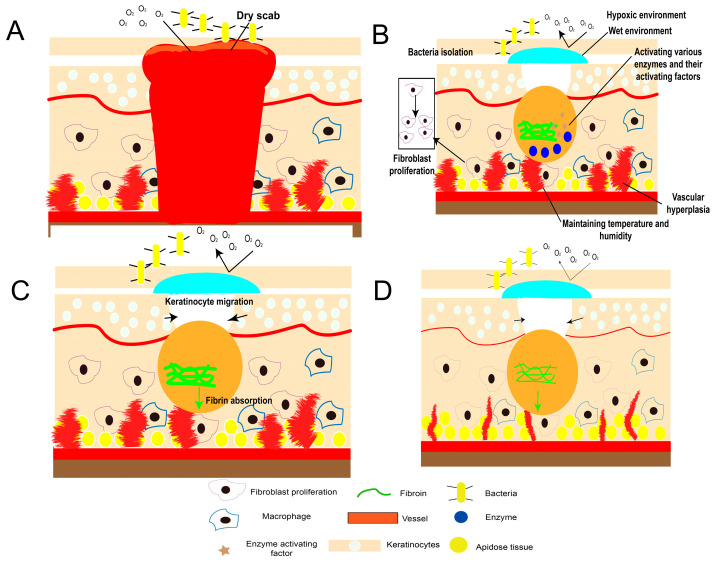
Wet healing theory: specific wet microenvironment can promote wound healing. (**A**) Initial inflammatory stage; (**B**) wet microenvironment protection; (**C**) keratinocyte migration; (**D**) regeneration outcome.

The theories about the local environment needed for wound healing mainly include dry healing theory and wet healing theory. For many years, dry healing theory has been the mainstream guiding theory in clinical treatment. This theory presumes that wound healing requires the participation of atmospheric oxygen and a dry environment. Nevertheless, one study shows that atmospheric oxygen cannot be directly used by the wound tissue, and thus it has no practical significance for wound healing, and excessive oxygen even hinders wound healing [13]. At the same time, the dry healing environment makes the wound easy to dehydrate and scab, which is not conducive to the crawling of epithelial cells. The growth factors secreted by the body will lose their biological activity easily, resulting in slow healing [14]. In addition, the dry environment cannot prevent bacterial invasion or maintain the humidity and temperature of the wound, which is not favorable for wound healing. Wet healing theory considers that a specific wet microenvironment is necessary for the process of wound healing. Its principles include (Figure 2): (1) Reducing the oxygen pressure of the wound. The hypoxic healing environment is conducive to the formation of epithelial cells and collagen fibers, promoting the growth of fibroblasts, stimulating vascular proliferation, and improving the stability of growth factors [15]. (2) Maintaining a certain temperature and humidity of the wound can retain the release of wound exudates, and activate a variety of enzymes and enzyme activation factors (especially protease and urokinase). These enzymes can promote the degradation and absorption of necrotic tissue and fibrin, and avoid the formation of scabs [16]. (3) Avoiding air exposure to nerve endings can reduce pain. (4) Accelerating the migration of epithelized epidermal cells rapidly can shrink the wound [17], increase the formation of granulation tissue, and re-epithelize the wound [18]. (5) The hypoxic and slightly acidic environment, with pH ranging from 5.0–6.0 under closed conditions, can directly inhibit the growth of bacteria [19], which is favorable for the reproduction and function of leukocytes to prevent the penetration of bacteria and improve local immunity [20].

At present, the wet healing theory is preferably accepted by the academic community, compared to the dry healing theory. Some traditional wound dressing components like polysaccharides and vaseline are used to ensure that the wet conditions can be applied to the treatment [21,22]. Researchers have been devoted to developing new wound dressings, as well. For example, Rodrigues et al. developed a wound dressing using a hydrocellular functional material, which was found to be effective in diabetic wound healing. The material has shown good results due to the excellent balance of exudates removal, and a moist film on the wound can prevent the formation of dry scab and facilitate epithelialization of wound [23]. Agarwal et al. found that a curcumin-loaded polycaprolactone/polyvinyl alcohol-silk fibroin based electrospun nanofibrous mat can provide a moist microenvironment, to protect the diabetic wound from secondary infections, remove the wound exudate, control the biofilm, and promote tissue regeneration [24]. Based on these studies, an ideal delivery system for chronic wound application should be able to form a uniform and well-sealed moist film on the wound surface and create a moist and hypoxic local environment.

#### 1.2.2. Sustained Delivery of Growth Factor

Growth factors are a class of bioactive proteins that significantly regulate cell growth and differentiation, which play important roles in the process of wound healing [25]. They have high efficiency and pleiotropy that can affect the processes of cell proliferation, migration, and extracellular matrix synthesis by regulating cell responses in the process of wound repair [26]. Although there have been many clinical trials on growth factor reparations, they were relatively small and single-centered. The results showed that the applications of growth factors to the intervention treatment of chronic wounds caused by burns, diabetic foot, and pressure ulcer can significantly accelerate the process of wound healing and improve the quality of healing [27,28,29]. The most widely used growth factor is human epidermal growth factor (hEGF). In addition, platelet-derived growth factor (PDGF) [30,31], basic fibroblast growth factor (bFGF) [32], and VEGF, have a considerable amount of applications [33].

The inactivation of these growth factors can be caused by different reasons, including physical changes such as salting-out and surface adsorption or chemical changes like breakage of bonds. Generally, chemical changes are the most important cause. Peptide bonds hydrolyze when the pH changes, while peptides are prone to aggregation at the isoelectric point. Temperature can cause changes in the conformation of peptides. As previously mentioned, different degradation reactions dominate under diverse conditions (such as temperature, pH, fluorescence, etc.) [34].

Specifically, an ideal delivery system to treat chronic wounds needs to have the following features: (1) Providing a moist and hypoxic environment suitable for wound healing; (2) releasing growth factors slowly and improving their stability [35]. In addition to these key attributes, it is expected to reduce the frequency of administration, be convenient to administrate, and finally improve the patient’s compliance.

## 2. Growth Factor Delivery Systems to Heal Chronic Wounds

As previously discussed, both suitable healing environment and stable growth factor delivery are considered as prerequisites and crucial for wound healing. In this section, existing and innovative delivery systems are evaluated according to the two preconditions.

### 2.1. Existing Delivery Systems

In recent years, a variety of growth factor delivery systems for chronic wounds have been developed, most of which are in the form of solution or gel, as listed in Table 1 [36]. The majority of these delivery systems need to be applied and replaced frequently, a situation which is prone to cause pain and increase the probability of wound infection [37,38]. Solution and gel systems also have two critical bottlenecks—inability to maintain a suitable healing environment and stable growth factor delivery. After the solution is administered, because the wound is an open system and the drug could not be retained on the wound for a long time, it is impossible to control the formation of a hypoxic and moist microenvironment, or the isolation and protection effect on the wound. The gel is a normally crosslinked structure formed by the interlacing of polymer chains [39,40], which is not strong to control the release of growth factor, and is prone to cracking or falling due to dehydration caused by changes in external environmental humidity [41]. As a result, the clinical efficacy of current growth factor delivery systems for chronic wounds available in the market at present, is far from being ideal.

### 2.2. Innovative Delivery Systems

**Figure 3:**
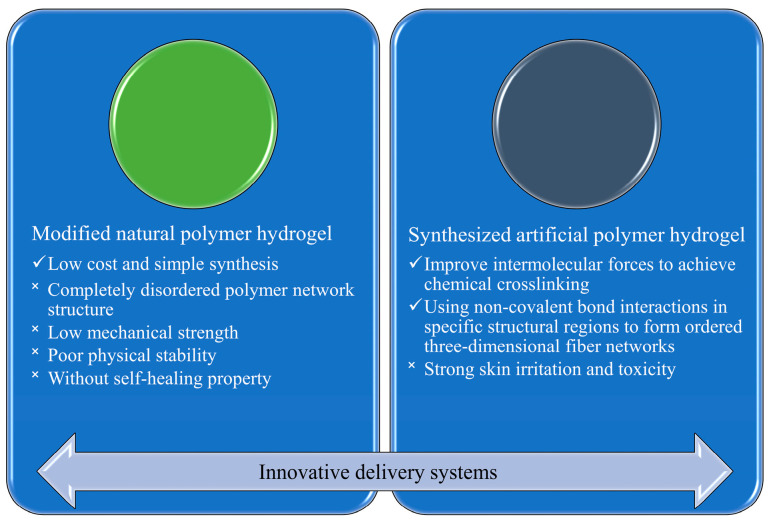
Comparison of two innovative delivery systems.

New delivery systems need to be developed to provide a suitable healing environment and stable growth factor delivery. Modified natural polymer hydrogel and synthesized artificial polymer hydrogel are possible systems (Figure 3), as discussed below.

#### 2.2.1. Hydrogels of Modified Natural Polymers

In order to provide a wet and hypoxic local environment suitable for wound healing, researchers have tried to use hydrogels that comprise natural polymers as drug carriers, such as sodium alginate, carrageenan, etc., which possess good biocompatibility and biodegradability and are less likely to cause immune rejection [42,43,44]. However, normal hydrogel possesses completely disordered polymeric network structures, with low mechanical strength, poor physical stability, and lack of self-healing property. It is difficult to maintain a hypoxic and moist environment for a wound consistently to meet the expectations of clinical use [45]. At the same time, this disordered network also has a limited stabilizing effect on polypeptide drugs such as growth factors. A sustained growth factor delivery cannot be obtained. Thus, investigators have improved the mechanical properties of natural polymer hydrogels by adding small organic molecules such as acrylamide and acrylic acids [46,47]. These molecules can react with the key functional groups of hydrogel components to form polymeric structures more orderly and create an anoxic and humid environment [48]. For example, Zhang et al. developed a new antibacterial hydrogel wound dressing that comprises poly(aminoethyl) modified chitosan, which not only can enhance its antibacterial activity, but also promote the formation and stabilization of the prepared hydrogel [49]. However, it is possible that the modification of natural polymers can lead to unexpected changes in biocompatibility when molecules react with functional groups or other functional groups are introduced in gels.

#### 2.2.2. Hydrogels of Synthesized Polymers

Scientists have also tried to prepare hydrogels with chemically synthesized polymers. Usually, crosslinking agents, initiators, chelators, and chain transfer agents such as glutaraldehyde, N,N-methylene bis-acrylamide, metal ions, and ammonium persulfate, respectively, need to be added at the same time to control the polymerization kinetics, but the residue of the above substances normally causes unwanted strong skin irritation and toxicity [50,51]. Some studies have also achieved crosslinking by increasing intermolecular forces such as hydrogen bonds and electrostatic coupling, and used noncovalent bonding in specific structural regions to make the polymers self-assemble into an orderly three-dimensional fiber network [52,53]. Specifically, green chemical crosslinking methods like free-radical polymerization, reaction of complementary groups, and enzymatic reaction can be applied in various hydrogel systems, considering the biochemical features of the polymers [54,55]. For example, Raia et al. developed a composite hydrogel in which hyaluronic acid was covalently crosslinked with silk, and silk themselves crosslinked via the effect of horseradish peroxidase. This kind of hydrogel exhibited both mechanical integrity and hydrophilicity [56]. Synthetic hydrogels are very different from natural polymer hydrogels in the chemical composition and preparation process, which affect the mechanical properties and stability of gels [57].

## 3. Lyotropic Liquid Crystalline Might Be a Promising Candidate for Chronic Wound Healing

Although hydrogels of modified natural polymers and synthesized polymers are applied to provide a suitable environment and stable growth factor delivery, these two strategies may introduce byproducts that provoke safety issues and limit their clinical use [58]. Therefore, a better new delivery system should be developed. Lyotropic liquid crystalline (LLC) is an ideal candidate for chronic wound healing with its properties of good biosafety, ease of industrialization, and low cost [59,60].

**Figure 4:**
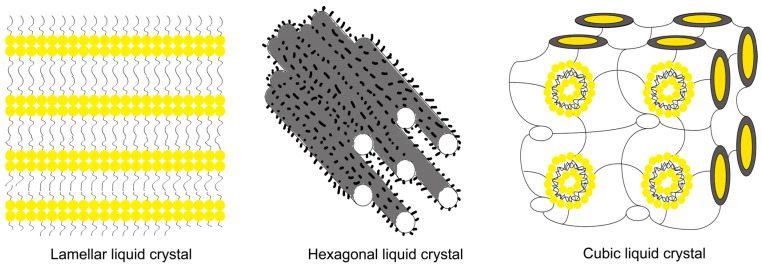
Forms of lyotropic liquid crystalline.

LLC is a long-range ordered liquid crystal structure formed by self-assembly of amphiphilic molecules and solvents (Figure 4). There are two common types of LLC. One kind is prepared from alkyl-based materials like fatty acid salts, alkyl sulfonates, etc. The hydrophilic parts of these materials such as carboxyl and sulfonic groups are linked to a long hydrophobic group, forming a polar “head” and two hydrophobic “tails”. The other type is prepared from phosphor-lipids such as egg/bean phospholipids and glycosphingolipids, with one polar “head” and two hydrophobic “tails” in the molecule. The hydrophobic groups in the molecule are usually arranged side-by-side [61]. Due to its spontaneous phase transition characteristics, ordered lattice structure, excellent drug loading, good biosafety and biocompatibility [62,63], release profile and stabilization of various drugs [64], it is expected to act as a prospect candidate delivery system for chronic wound treatment. LLC not only has a healing effect on the wound, but also can be useful as a nano drug carrier in implants [65,66].

LLC was featured with a unique spontaneous transition process from low-viscosity precursor to high-viscosity gel triggered by medium change. Its precursors are generally a layered liquid crystalline, which is formed by the superposition of bimolecular layers that comprise amphiphilic molecules, contributing to good fluidity. The polypeptide drugs (like growth factors) can be incorporated in the interlayers between the bimolecular layers. The precursors can be evenly smeared or sprayed on the wound surface when contacted with water, then a gel with ordered lattice structure is rapidly formed, transitioning from a layered phase to a cubic phase. Herein, a relatively closed aqueous channel is formed for loading growth factors and the formed LLC fits the non-smooth wound surface. Moreover, a hypoxic environment is created by the physical occultation, which can avoid the degradation of encapsulated drugs in contact with external water and oxygen molecules [67].

### 3.1. LLC Can Provide Sustained and Stable Growth Factor Delivery

**Figure 5:**
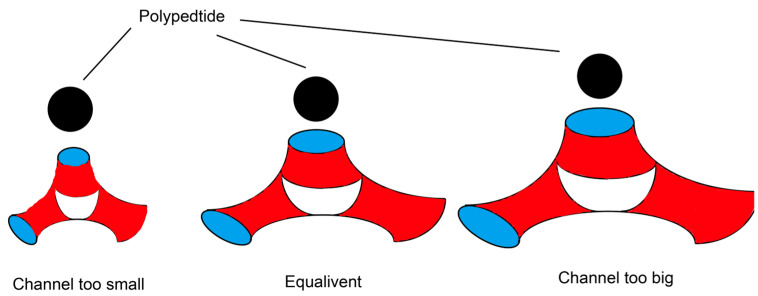
Relationship between the size of water channel and polypeptide to be embedded.

If the diameter of the water channel is smaller than the molecular size of the polypeptide, the polypeptide cannot be completely encapsulated in the water channel, which may result in irregular drug release behavior and degradation [68]. In contrast, if the water channel is larger than the polypeptide, the effect of LLC on improving the stability of polypeptide drugs weakens. Only if the size of the water channel is similar to that of the polypeptide, the LLC lattice structure can embed the polypeptide nicely in the water channel, and the controlled release and stability of polypeptide can be expected. It is speculated that the size of the water channel can be adjusted to match the molecular size of the growth factors by optimizing the formulation, in order to improve the stability of the growth factors in the LLC system (Figure 5) [69].

The release of drug from LLC is generally a concentration-mediated diffusion process. Specifically, the drug with higher concentrations in liquid crystalline carrier diffuses into the release medium with low concentrations. In general, the release of drugs from LLC conforms to Higuchi equation (Equation (1)) or Fick diffusion equation (Equation (2)) [70,71]:

(1) $$\frac{Q}{A}=2C_{0}\sqrt{\frac{Dt}{\pi }}$$

where *Q*/*A* represent the amount of drug diffusing into the receiving tank per unit diffusion area, *C*_0_ is the initial drug concentration, *D* represents the drug apparent diffusion coefficient, and *t* means diffusion time of drug:

(2) $$dS=-DF\frac{dC}{dx}\times dt$$

where *dS* represents the diffusion of substance (solute) in *dt* time, *D* is the diffusion coefficient, *F* is the diffusion area, and *dC*/*dx* represents the concentration gradient.

It is conjectured that through the sequential collapse of the lattice structure of LLC, the release rate of growth factor can be precisely regulated and the release mechanisms can be explained by Higuchi equation or Fick diffusion equation.

### 3.2. LLC Can Provide a Suitable Healing Environment for Wounds

Three major advantages of LLC can provide a suitable healing environment.

First, LLC can promote cell adhesion and proliferation. Its bimolecular layers comprise amphiphilic molecules that can provide a microenvironment similar to the cell membrane. Under physiological conditions, this biomimetic structure has good affinity and compatibility with the wound tissue [72], and acts as a skeleton or platform for tissue regeneration.

Second, LLC can provide a moist local environment for the wound. The water containing capacity of the system is due to its specific lattice structure. Common hydrogels (such as sodium alginate gel) lack a lattice structure [73], while the water channels within the LLC lattice structure have better water retention. The ordered lattice structure maintains a local environment with a certain temperature and humidity for the wound, which is favorable to activate a variety of enzymes and enzyme-activating factors, and promote the degradation and absorption of necrotic tissue and fibrin. In addition, the gel with certain mechanical strength formed can closely adhere to the uneven wound surface. LLC has low-oxygen permeability to isolate the external oxygen and reduce the oxygen pressure in the wound [74]. Nevertheless, the degradation products of LLC like glycerol have a moisturizing effect and oleic acid is non-irritating to the skin [75].

**Figure 6:**
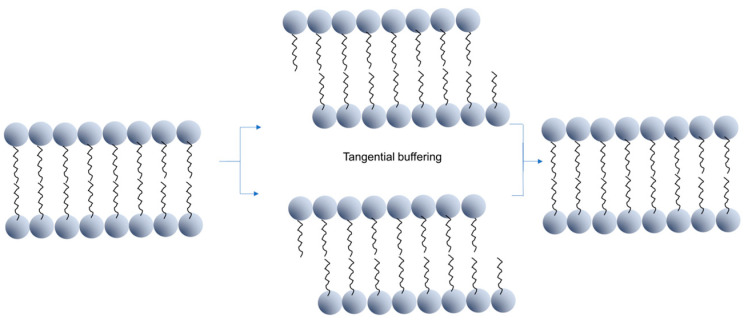
Bimolecular layers in the lyotropic liquid crystal exhibit the spring-like effect.

Third, LLC is a viscoelastic material. Upon the application of an external force, the lipid bilayer in the lattice structure has a spring-like effect, viz., it can respond to the external force by generating molecular compression and lattice rearrangement, and restore the original ordered structure. This effectively buffers and absorbs the impact force on the wound and protects the wound (Figure 6) [76]. Of note, the mechanical strength of LLC can be altered by the lattice structure. By controlling the lattice structure, the mechanical strength of the system can be adjusted. In summary, LLC not only can deliver the growth factor sustainably, but also maintain an environment that is beneficial for chronic wound healing. We call on more researchers to devote to this research and accelerate its translation and application.

### 3.3. LLC Application in Wound Healing

LLC has made some progress as a wound healing dressing system, listed in Table 2. Zhou et al. constructed recombinant human epidermal growth factor (rhEGF)-containing lyotropic liquid crystalline precursor systems for chronic wound therapy, which exhibit good cargo stability and mechanical properties. It showed distinct promotion effects on wound closure, inflammatory recovery, and re-epithelization process in Sprague–Dawley rat models. Interestingly, different diameters of the internal water channels led to different in vitro release rates of rhEGF, and finally different therapeutic responses in cellulo and in vivo. Yue et al. investigated an LLC-based bacteria-resistant and self-healing spray dressing loaded with ε-polylysine, which can kill antibiotic-resistant bacteria efficiently, even the methicillin-resistant Staphylococcus aureus. The cubic cells of LLC could encapsulate PLL to improve its stability and induce a sustained release. The LLC precursor could spontaneously transit to a cubic phase gel once exposed to physiological fluid, which is endowed with mechanically responsive viscoelasticity that builds a robust and flexible defense for wounds. Chen et al. designed a hyaluronic acid combined LLC-based spray dressing loaded with the anti-fibrotic drug pirfenidone. The dressing possessed high levels of water absorption for exudate absorption. The self-assembled lattice nanostructures provide excellent mechanical protection to promote the healing process and steady pirfenidone release to exert a scar prophylaxis effect. In the deep partial thickness burn wound model they established, the dressing performs excellent healing effects, and pirfenidone-loaded dressing displayed an ideal prognosis with skin as smooth as healthy skin. The healing promotion of dressing was considered to be related to a clearly shortened inflammation phase, with contributions from water management and mechanical protection by the dressing. The scar prophylaxis of pirfenidone-loaded dressing was proven to be related to the regulation of collagen synthesis and degradation. It offered significant promise as a spray dressing for deep partial thickness burn injuries.

## 4. Conclusions and Future Directions

Treatment of chronic wound is a serious health issue that is expanding around the world. Considering the different wound healing stages, providing a suitable healing environment and stable growth factor delivery are the keys to the treatment of chronic wounds. Existing delivery systems are difficult to meet all the requirements. Delivery systems like modified natural polymer hydrogel and synthesized artificial polymer hydrogel have been applied to heal chronic wounds. For natural polymers, small organic molecules can modify the gels to increase mechanical strength. Hydrogels that comprise chemically synthesized polymers can minimize their skin toxicity by green chemical crosslinking. However, they still have problems in biosafety and industrialization. Thus, there is a critical and urgent need to develop new systems which are able to fulfill the above demands. As an innovative delivery system, LLC meets the requirements of chronic wound healing. It builds a moist environment and its structure can keep the structure and activity of growth factors stable. The release of growth factor can be regulated in the LLC gel. Additionally, it shows advantages such as biocompatibility over other systems, which has a promising future for clinical application.

The research on wound healing has been proceeding rapidly. Numerous efforts are put into the studies of wound pathogenesis and providing insights about the mechanisms of healing process. Moreover, advances in pharmaceutical sciences have resulted in the production of new active molecules that can improve the tissue regeneration rate and accelerate the compromised physiologic processes. Along with these advances, more reliable and applicable drug delivery systems will be designed and tested. The current wound healing preparations still have many limitations, mainly in manufacturing. An ideal wound healing product should be stable and sterile. It is not easy to maintain the activity of ingredients and maintain an aseptic state at the same time, especially for protein drugs. In addition, the manual labor required for synthesis and raw material cost should be considered [80,81]. In the future, drug delivery systems for chronic wound applications will be expected to be commercially developed through different aspects, such as simplifying the preparation methods and establishing quality control of the final products.

## Figures and Tables

**Table 1:** Commercial formulations of growth factor drugs that are currently widely used in the clinic [36].

Growth Factor	Number of Amino Acid Residue	Commercial/Clinical Products for Wound Treatment	Dosage Form	Matrix Material
Epidermal growth factor	53	HEBERPROT-P^®^ (Heber, Biotech, Havana, Cuba) EASYEF^®^ (Daewoong Pharmaceutical, Seoul, Republic of Korea) REGEN-D^TM^150 (Bharat Biotech International Limited, Hyderabad, India)	Solution Solution Gel	Normal saline Normal saline —
Platelet-derived growth factor	196–370	Regranex^®^ (Smith and Nephew, Inc., London, UK)	Gel	Carboxymethyl cellulose
Basic fibroblast growth factor	155	Fiblast^®^Spray (Kaken Pharmaceutical Co., Ltd., Tokyo, Japan)	Solution	Normal saline
Vascular endothelial growth factor	121–206	Telbermin (Genentech, South San Francisco, CA, USA), phase I	Gel	—

**Table 2:** Representing articles about wound healing using LLC.

No.	Main Materials	Type of Wound	Loading Drug	Reference
1	GMO	Chronic wound	rhEGF	[77]
2	GMO	Post-operative wound	ε-polylysine	[78]
3	Hyaluronic acid, GMO	Burn wound	Pirfenidone	[79]

## Data Availability

Not applicable.

## References

[1] Powers J.G., Higham C., Broussard K., Phillips T.J. (2016). Wound healing and treating wounds: Chronic wound care and management. J. Am. Acad. Dermatol..

[2] Maxson S., Lopez E.A., Yoo D., Danilkovitch-Miagkova A., LeRoux M.A. (2012). Concise review: Role of mesenchymal stem cells in wound repair. Stem Cells Transl. Med..

[3] Skrepnek G.H., Mills J.L., Lavery L.A., Armstrong D.G. (2017). Health care service and outcomes among an estimated 6.7 million ambulatory care diabetic foot cases in the US. Diabetes Care.

[4] Zhao S., Li L., Wang H., Zhang Y., Cheng X., Zhou N., Rahaman M.N., Liu Z., Huang W., Zhang C. (2015). Wound dressings composed of copper-doped borate bioactive glass microfibers stimulate angiogenesis and heal full-thickness skin defects in a rodent model. Biomaterials.

[5] Tottoli E.M., Dorati R., Genta I., Chiesa E., Pisani S., Conti B. (2020). Skin wound healing process and new emerging technologies for skin wound care and regeneration. Pharmaceutics.

[6] Ellis S., Lin E.J., Tartar D. (2018). Immunology of wound healing. Curr. Dermatol. Rep..

[7] Wang Z., Qi F., Luo H., Xu G., Wang D. (2022). Inflammatory microenvironment of skin wounds. Front. Immunol..

[8] Xiang Y., Qi X., Cai E., Zhang C., Wang J., Lan Y., Deng H., Shen J., Hu R. (2023). Highly efficient bacteria-infected diabetic wound healing employing a melanin-reinforced biopolymer hydrogel. Chem. Eng. J..

[9] Bayat M., Sarojini H., Chien S. (2023). The role of cluster of differentiation 163-positive macrophages in wound healing: A preliminary study and a systematic review. Arch. Dermatol. Res..

[10] Li L., Ma Y., He G., Ma S., Wang Y., Sun Y. (2023). Pilose antler extract restores type I and III collagen to accelerate wound healing. Biomed. Pharmacother..

[11] Belvedere R., Novizio N., Morello S., Petrella A. (2022). The combination of mesoglycan and VEGF promotes skin wound repair by enhancing the activation of endothelial cells and fibroblasts and their cross-talk. Sci. Rep..

[12] Spielman A.F., Griffin M.F., Parker J., Cotterell A.C., Wan D.C., Longaker M.T. (2023). Beyond the Scar: A Basic Science Review of Wound Remodeling. Adv. Wound Care.

[13] Stahl H.C., Ahmadi F., Nahzat S.M., Dong H.J., Stahl K.W., Sauerborn R. (2018). Health economic evaluation of moist wound care in chronic cutaneous leishmaniasis ulcers in Afghanistan. Infect. Dis. Poverty.

[14] Liu Y.S., Men J., Zhang L.T., Cheng L.F., Yang W.B., Zhang W.H. (2015). Microstructural evolution and self-healing mechanism of a 2D C/SiC-BCx composite under constant load in static wet oxygen and dynamic combustion atmosphere. Mater. Corros..

[15] Steiner C.A., Cartwright I.M., Taylor C.T., Colgan S.P. (2022). Hypoxia-inducible factor as a bridge between healthy barrier function, wound healing, and fibrosis. Am. J. Physiol.—Cell Physiol..

[16] Skórkowska-Telichowska K., Czemplik M., Kulma A., Szopa J. (2013). The local treatment and available dressings designed for chronic wounds. J. Am. Acad. Dermatol..

[17] Luan X., Li W., Lou F. (2016). Applied analysis of humanized nursing combined with wet healing therapy to prevent bedsore. Eur. Rev. Med. Pharmacol. Sci..

[18] Zahid M., Lodhi M., Rehan Z.A., Tayyab H., Javed T., Shabbir R., Mukhtar A., El Sabagh A., Adamski R., Sakran M.I. (2021). Sustainable development of chitosan/Calotropis procera-based hydrogels to stimulate formation of granulation tissue and angiogenesis in wound healing applications. Molecules.

[19] Han X., Ju L.S., Irudayaraj J. (2023). Oxygenated Wound Dressings for Hypoxia Mitigation and Enhanced Wound Healing. Mol. Pharm..

[20] Han S.-K. (2023). Innovations and Advances in Wound Healing.

[21] Lei H., Zhu C., Fan D. (2020). Optimization of human-like collagen composite polysaccharide hydrogel dressing preparation using response surface for burn repair. Carbohydr. Polym..

[22] Wang X.-F., Li M.-L., Fang Q.-Q., Zhao W.-Y., Lou D., Hu Y.-Y., Chen J., Wang X.-Z., Tan W.-Q. (2021). Flexible electrical stimulation device with Chitosan-Vaseline^®^ dressing accelerates wound healing in diabetes. Bioact. Mater..

[23] Rodrigues M., Govindharajan T. (2021). Study of hydrocellular functional material as microbicidal wound dressing for diabetic wound healing. J. Appl. Biomater. Funct. Mater..

[24] Agarwal Y., Rajinikanth P., Ranjan S., Tiwari U., Balasubramnaiam J., Pandey P., Arya D.K., Anand S., Deepak P. (2021). Curcumin loaded polycaprolactone-/polyvinyl alcohol-silk fibroin based electrospun nanofibrous mat for rapid healing of diabetic wound: An in-vitro and in-vivo studies. Int. J. Biol. Macromol..

[25] Zarei F., Soleimaninejad M. (2018). Role of growth factors and biomaterials in wound healing. Artif. Cells Nanomed. Biotechnol..

[26] Cheng B., Yan Y., Qi J., Deng L., Shao Z.-W., Zhang K.-Q., Li B., Sun Z., Li X. (2018). Cooperative assembly of a peptide gelator and silk fibroin afford an injectable hydrogel for tissue engineering. ACS Appl. Mater. Interfaces.

[27] Gragnani A., Tonarelli E., Chomiski V., Daher R.P., Ferreira L. (2022). Fibroblast growth factor in the treatment of burns: A systematic review. Burns.

[28] Kumar N., Verma A., Mishra A., Agrawal G., Agrawal A., Mishra S. Platelet Derived Growth Factor in Healing of Large Diabetic Foot Ulcers in Indian Clinical Set-Up: A Protocol-Based Approach. http://static.webmedcentral.com/article_view/3985.

[29] Landi F., Aloe L., Russo A., Cesari M., Onder G., Bonini S., Carbonin P.U., Bernabei R. (2003). Topical treatment of pressure ulcers with nerve growth factor: A randomized clinical trial. Ann. Intern. Med..

[30] Papanas D., Maltezos E. (2010). Benefit-risk assessment of becaplermin in the treatment of diabetic foot ulcers. Drug Saf..

[31] Ziyadeh N., Fife D., Walker A.M., Wilkinson G.S., Seeger J.D. (2011). A matched cohort study of the risk of cancer in users of becaplermin. Adv. Ski. Wound Care.

[32] Uchi H., Igarashi A., Urabe K., Koga T., Nakayama J., Kawamori R., Tamaki K., Hirakata H., Ohura T., Furue M. (2009). Clinical efficacy of basic fibroblast growth factor (bFGF) for diabetic ulcer. Eur. J. Dermatol..

[33] Goswami A.G., Basu S., Huda F., Pant J., Ghosh Kar A., Banerjee T., Shukla V.K. (2022). An appraisal of vascular endothelial growth factor (VEGF): The dynamic molecule of wound healing and its current clinical applications. Growth Factors.

[34] Lee K., Silva E.A., Mooney D.J. (2011). Growth factor delivery-based tissue engineering: General approaches and a review of recent developments. J. R. Soc. Interface.

[35] Zhang X., Feng J., Feng W., Xu B., Zhang K., Ma G., Li Y., Yang M., Xu F.-J. (2022). Glycosaminoglycan-based hydrogel delivery system regulates the wound microenvironment to rescue chronic wound healing. ACS Appl. Mater. Interfaces.

[36] Lau H.-C., Kim A. (2016). Pharmaceutical perspectives of impaired wound healing in diabetic foot ulcer. J. Pharm. Investig..

[37] Garcia-Orue I., Gainza G., Gutierrez F.B., Aguirre J.J., Evora C., Pedraz J.L., Hernandez R.M., Delgado A., Igartua M. (2017). Novel nanofibrous dressings containing rhEGF and Aloe vera for wound healing applications. Int. J. Pharm..

[38] Xia G., Liu Y., Tian M., Gao P., Bao Z., Bai X., Yu X., Lang X., Hu S., Chen X. (2017). Nanoparticles/thermosensitive hydrogel reinforced with chitin whiskers as a wound dressing for treating chronic wounds. J. Mater. Chem. B.

[39] Khan M.U.A., Stojanović G.M., Hassan R., Anand T.J.S., Al-Ejji M., Hasan A. (2023). Role of Graphene Oxide in Bacterial Cellulose− Gelatin Hydrogels for Wound Dressing Applications. ACS Omega.

[40] Al-Arjan W.S., Khan M.U.A., Almutairi H.H., Alharbi S.M., Razak S.I.A. (2022). pH-Responsive PVA/BC-f-GO dressing materials for burn and chronic wound healing with curcumin release kinetics. Polymers.

[41] Li J.-Y., Lin Y.-T., Wang D.K., Tseng H.-H., Wey M.-Y. (2023). Planetary cross-linked structure design of hybrid organosilica membrane by amine-driven polymerization for CO_2_ separation. J. Clean. Prod..

[42] Williams P.A., Campbell K.T., Silva E.A. (2018). Alginate hydrogels of varied molecular weight distribution enable sustained release of sphingosine-1-phosphate and promote angiogenesis. J. Biomed. Mater. Res. Part A.

[43] Park H.-H., Ko S.-C., Oh G.-W., Jang Y.-M., Kim Y.-M., Park W.S., Choi I.-W., Jung W.-K. (2018). Characterization and biological activity of PVA hydrogel containing chitooligosaccharides conjugated with gallic acid. Carbohydr. Polym..

[44] Zepon K.M., Marques M.S., da Silva Paula M.M., Morisso F.D.P., Kanis L.A. (2018). Facile, green and scalable method to produce carrageenan-based hydrogel containing in situ synthesized AgNPs for application as wound dressing. Int. J. Biol. Macromol..

[45] Wang Z., An G., Zhu Y., Liu X., Chen Y., Wu H., Wang Y., Shi X., Mao C. (2019). 3D-printable self-healing and mechanically reinforced hydrogels with host–guest non-covalent interactions integrated into covalently linked networks. Mater. Horiz..

[46] Peppas N.A. (1997). Hydrogels and drug delivery. Curr. Opin. Colloid Interface Sci..

[47] Sennakesavan G., Mostakhdemin M., Dkhar L., Seyfoddin A., Fatihhi S. (2020). Acrylic acid/acrylamide based hydrogels and its properties—A review. Polym. Degrad. Stab..

[48] Zhang X., Qin M., Xu M., Miao F., Merzougui C., Zhang X., Wei Y., Chen W., Huang D. (2021). The fabrication of antibacterial hydrogels for wound healing. Eur. Polym. J..

[49] Zhang Y., Dang Q., Liu C., Yan J., Cha D., Liang S., Li X., Fan B. (2017). Synthesis, characterization, and evaluation of poly (aminoethyl) modified chitosan and its hydrogel used as antibacterial wound dressing. Int. J. Biol. Macromol..

[50] Hoffman A.S. (2012). Hydrogels for biomedical applications. Adv. Drug Deliv. Rev..

[51] Wang Y. (2015). Swelling behavior of konjac glucomannan/N, n-dimethylene bisacrylamide hydrogel. Anhui Agron. Bull..

[52] Wang Y., Zhang B., Ma M., Lu W. (2017). Preparation of gelma/PEGDA hydrogel by UV copolymerization and crosslinking. Imaging Sci. Photochem..

[53] Wang Q., Ren L., Wang Y., Yao Y. (2013). Preparation and characterization of photocrosslinked n-acryloyl glucosamine/PEGDA hydrogel. J. South China Univ. Technol..

[54] Su J., Satchell S.C., Wertheim J.A., Shah R.N. (2019). Poly (ethylene glycol)-crosslinked gelatin hydrogel substrates with conjugated bioactive peptides influence endothelial cell behavior. Biomaterials.

[55] Wu X., He C., Wu Y., Chen X. (2016). Synergistic therapeutic effects of Schiff’s base cross-linked injectable hydrogels for local co-delivery of metformin and 5-fluorouracil in a mouse colon carcinoma model. Biomaterials.

[56] Raia N.R., Partlow B.P., McGill M., Kimmerling E.P., Ghezzi C.E., Kaplan D.L. (2017). Enzymatically crosslinked silk-hyaluronic acid hydrogels. Biomaterials.

[57] Gyles D.A., Castro L.D., Silva Jr J.O.C., Ribeiro-Costa R.M. (2017). A review of the designs and prominent biomedical advances of natural and synthetic hydrogel formulations. Eur. Polym. J..

[58] Al-Tabakha M.M., Khan S.A., Ashames A., Ullah H., Ullah K., Murtaza G., Hassan N. (2021). Synthesis, characterization and safety evaluation of sericin-based hydrogels for controlled delivery of acyclovir. Pharmaceuticals.

[59] Cao Y., Wang P.X., D’Acierno F., Hamad W.Y., Michal C.A., MacLachlan M.J. (2020). Tunable diffraction gratings from biosourced lyotropic liquid crystals. Adv. Mater..

[60] Ye T.-J., Qian S., Gao Y., Yu M.-J., Wei Y.-F. (2019). Research Status, Problems and Countermeasures of Lyotropic Liquid Crystal in New Drug Delivery Systems of Traditional Chinese Medicine. Chin. J. Exp. Tradit. Med. Formulae.

[61] De Souza J.F., Pontes K.d.S., Alves T.F., Amaral V.A., Rebelo M.d.A., Hausen M.A., Chaud M.V. (2017). Spotlight on biomimetic systems based on lyotropic liquid crystal. Molecules.

[62] Shan X., Li X., Luo Z., Lin Q., Lu Y., Jiang M., Zhang J., Huang J., Xie L., Guo X. (2023). A Clinically-Achievable Injectable and Sprayable in Situ Lyotropic Liquid Crystalline Platform in Treating Hormone-Sensitive and Castration-Resistant Prostate Cancer. ACS Nano.

[63] Mancuso A., Cianflone E., Cristiano M.C., Salerno N., Tarsitano M., Marino F., Molinaro C., Fresta M., Torella D., Paolino D. (2022). Lyotropic liquid crystals: A biocompatible and safe material for local cardiac application. Pharmaceutics.

[64] Boyd B.J., Whittaker D.V., Khoo S.-M., Davey G. (2006). Lyotropic liquid crystalline phases formed from glycerate surfactants as sustained release drug delivery systems. Int. J. Pharm..

[65] Silvestrini A.V.P., Caron A.L., Viegas J., Praca F.G., Bentley M.V.L.B. (2020). Advances in lyotropic liquid crystal systems for skin drug delivery. Expert Opin. Drug Deliv..

[66] Jain S., Yadav P., Swami R., Swarnakar N.K., Kushwah V., Katiyar S.S. (2018). Lyotropic liquid crystalline nanoparticles of amphotericin B: Implication of phytantriol and glyceryl monooleate on bioavailability enhancement. AAPS PharmSciTech.

[67] Ibrahim T.M., El-Megrab N.A., El-Nahas H.M. (2021). An overview of PLGA in-situ forming implants based on solvent exchange technique: Effect of formulation components and characterization. Pharm. Dev. Technol..

[68] Das K., Roy B., Satpathi S., Hazra P. (2019). Impact of topology on the characteristics of water inside cubic lyotropic liquid crystalline systems. J. Phys. Chem. B.

[69] Negrini R., Mezzenga R. (2012). Diffusion, molecular separation, and drug delivery from lipid mesophases with tunable water channels. Langmuir.

[70] Ghanbari R., Assenza S., Saha A., Mezzenga R. (2017). Diffusion of polymers through periodic networks of lipid-based nanochannels. Langmuir.

[71] Meikle T.G., Yao S., Zabara A., Conn C.E., Drummond C.J., Separovic F. (2017). Predicting the release profile of small molecules from within the ordered nanostructured lipidic bicontinuous cubic phase using translational diffusion coefficients determined by PFG-NMR. Nanoscale.

[72] Rapalli V.K., Waghule T., Hans N., Mahmood A., Gorantla S., Dubey S.K., Singhvi G. (2020). Insights of lyotropic liquid crystals in topical drug delivery for targeting various skin disorders. J. Mol. Liq..

[73] Smidsrød O., Skja G. (1990). Alginate as immobilization matrix for cells. Trends Biotechnol..

[74] van ‘t Hag L., Li X., Meikle T.G., Hoffmann S.V., Jones N.C., Pedersen J.S., Hawley A.M., Gras S.L., Conn C.E., Drummond C.J. (2016). How peptide molecular structure and charge influence the nanostructure of lipid bicontinuous cubic mesophases: Model synthetic WALP peptides provide insights. Langmuir.

[75] Clapper J.D., Guymon C.A. (2007). Nanostructured biodegradable polymer composites generated using lyotropic liquid crystalline media. Macromolecules.

[76] Wang H., Peng T., Wu H., Chen J., Chen M., Mei L., Li F., Wang W., Wu C., Pan X. (2021). In situ biomimetic lyotropic liquid crystal gel for full-thickness cartilage defect regeneration. J. Control. Release.

[77] Zhou C., Huang Z., Huang Y., Wang B., Yang P., Fan Y., Hou A., Yang B., Zhao Z., Quan G. (2019). In situ gelation of rhEGF-containing liquid crystalline precursor with good cargo stability and system mechanical properties: A novel delivery system for chronic wounds treatment. Biomater. Sci..

[78] Yue X., Zhang X., Wang C., Huang Y., Hu P., Wang G., Cui Y., Xia X., Zhou Z., Pan X. (2021). A bacteria-resistant and self-healing spray dressing based on lyotropic liquid crystals to treat infected post-operative wounds. J. Mater. Chem. B.

[79] Chen J., Wang H., Mei L., Wang B., Huang Y., Quan G., Lu C., Peng T., Pan X., Wu C. (2020). A pirfenidone loaded spray dressing based on lyotropic liquid crystals for deep partial thickness burn treatment: Healing promotion and scar prophylaxis. J. Mater. Chem. B.

[80] Hou Y., Li J., Guan S., Witte F. (2021). The therapeutic potential of MSC-EVs as a bioactive material for wound healing. Eng. Regen..

[81] Akombaetwa N., Bwanga A., Makoni P.A., Witika B.A. (2022). Applications of Electrospun Drug-Eluting Nanofibers in Wound Healing: Current and Future Perspectives. Polymers.

